# Cranial-first approach of laparoscopic left colectomy for T4 descending colon cancer invading the Gerota’s fascia

**DOI:** 10.1186/s40792-019-0720-8

**Published:** 2019-10-28

**Authors:** Atsushi Ogura, Ryutaro Kobayashi, Satoru Kawai, Kenji Takagi, Kiyotaka Kawai, Takashi Maeda, Tsukasa Aritake, Natsuki Nagano, Satoaki Kamiya

**Affiliations:** Department of Surgery, Tsushima City Hospital, 3-73, Tachibana Town, Tsushima City, Aichi 496-8537 Japan

**Keywords:** T4 colon cancer, Cranial-first approach, Left colectomy

## Abstract

**Background:**

The safety and feasibility of laparoscopic colectomy for T4 colorectal cancer remain controversial. We believe that setting a “Goal” that will guide the surgeons in returning from the deep layer could be the key to safe en bloc resection of neighboring organs. For descending colon cancer, the cranial-first approach makes it possible to clearly visualize the pancreas and origin of the transverse mesocolon, leading to safe splenic flexure mobilization and complete mesocolic excision, which is the strongest advantage of this approach.

**Case presentation:**

A 75-year-old woman was diagnosed with T4 descending colon cancer invading the Gerota’s fascia. We performed laparoscopic left colectomy using the cranial-first approach to set a “Goal” at the inferior border of the pancreas for safe resection of the Gerota’s fascia. The total operative time was 233 min, and the estimated blood loss was 98 ml. She was discharged after surgery without postoperative complications. Pathological findings revealed the invasion into the Gerota’s fascia, and the resection margin was negative for cancer.

**Conclusions:**

The cranial-first approach of laparoscopic left colectomy appears to be safe and feasible and could be a promising method for selected patients with T4 descending colon cancer invading the Gerota’s fascia.

## Background

Laparoscopic colectomy for left-sided colon cancer is technically demanding owing to the complicated anatomy around the pancreas and transverse mesocolon [1]. For complete mesocolic excision (CME), dissection of the transverse mesocolon is necessary in the proximity of the pancreas. However, the thick mesocolon often prevents surgeons from detecting the pancreas, resulting in pancreatic injury or incomplete CME.

For left-sided colon cancer, the cranial-first approach is one of the promising procedures for obtaining better surgical outcomes; it has been reported previously [2]. This approach involves dissection of the superior layer of the mesocolon and transverse mesocolon at the posterior border of the pancreas cranially; this makes it possible to clearly visualize the pancreas and origin of the transverse mesocolon, leading to safe splenic flexure mobilization and CME, which is the strongest advantage of this approach.

For T4 descending colon cancer invading the Gerota’s fascia, dissection of the Gerota’s fascia is needed to obtain a sufficient surgical margin. The plane between the Gerota’s fascia and left kidney, which leads to the posterior layer of the pancreas, is relatively easy to detect. However, using the medial approach, it is difficult to return from this layer to the superior layer of the mesocolon because the thick Gerota’s fascia envelops the pancreas. The cranial-first approach would make it easier to detect the optimal layer in the medial approach, avoiding pancreatic injury and achieving CME.

Herein, we report the first case of cranial-first approach of laparoscopic left colectomy for T4 descending colon cancer invading the Gerota’s fascia.

## Case presentation

**Fig. 1:**
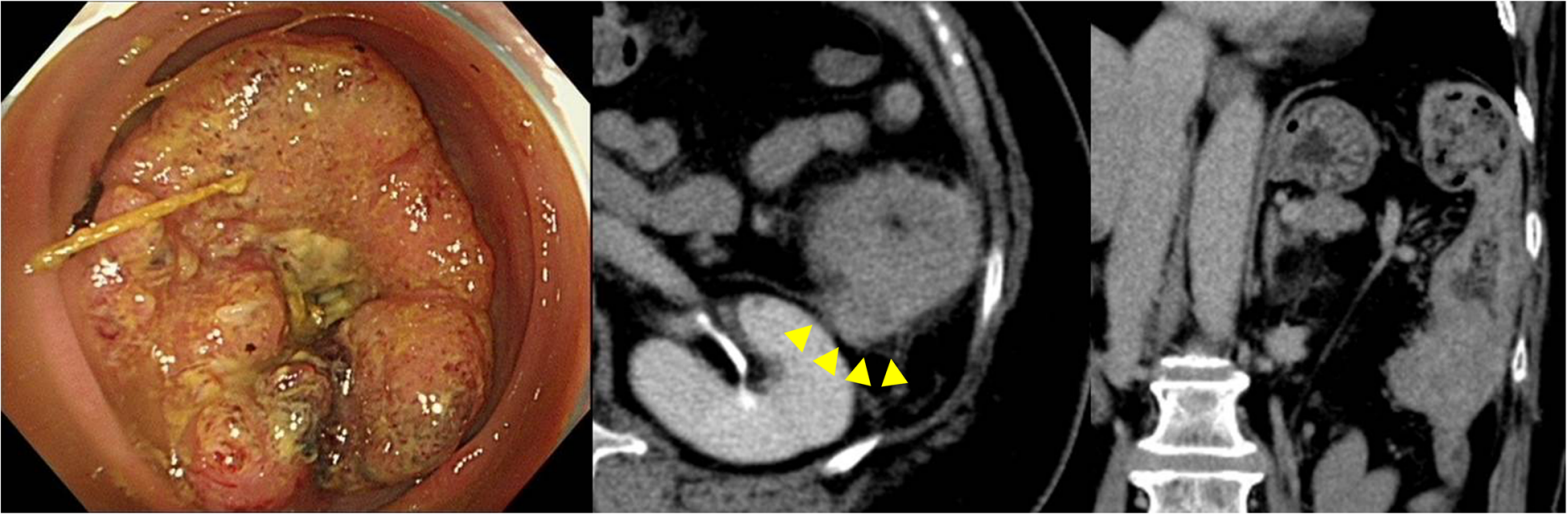
Colonoscopy and computed tomography: circumferential T4 descending colon cancer invading the Gerota’s fascia (arrow head) at the anal side of the splenic flexure

Written informed consent was obtained from the patient for publication of this case report, and her anonymity has been protected. A 75-year-old woman presenting with diarrhea was referred to our hospital. Colonoscopy showed a circumferential type II tumor located at the descending colon at the anal side of the splenic flexure. Histopathological examination revealed moderately differentiated adenocarcinoma. Computed tomography (CT) showed a 60 × 60-mm tumor located at the descending colon, invading the Gerota’s fascia (Fig. 1). Some swollen lymph nodes were detected along the left colic artery (LCA) and inferior mesenteric artery (IMA). No distant metastases were seen. Preoperative diagnosis was cT4bN1bM0 stage IIIC locally advanced descending colon cancer. We planned to perform laparoscopic left colectomy with en bloc resection of the Gerota’s fascia.

**Fig. 2:**
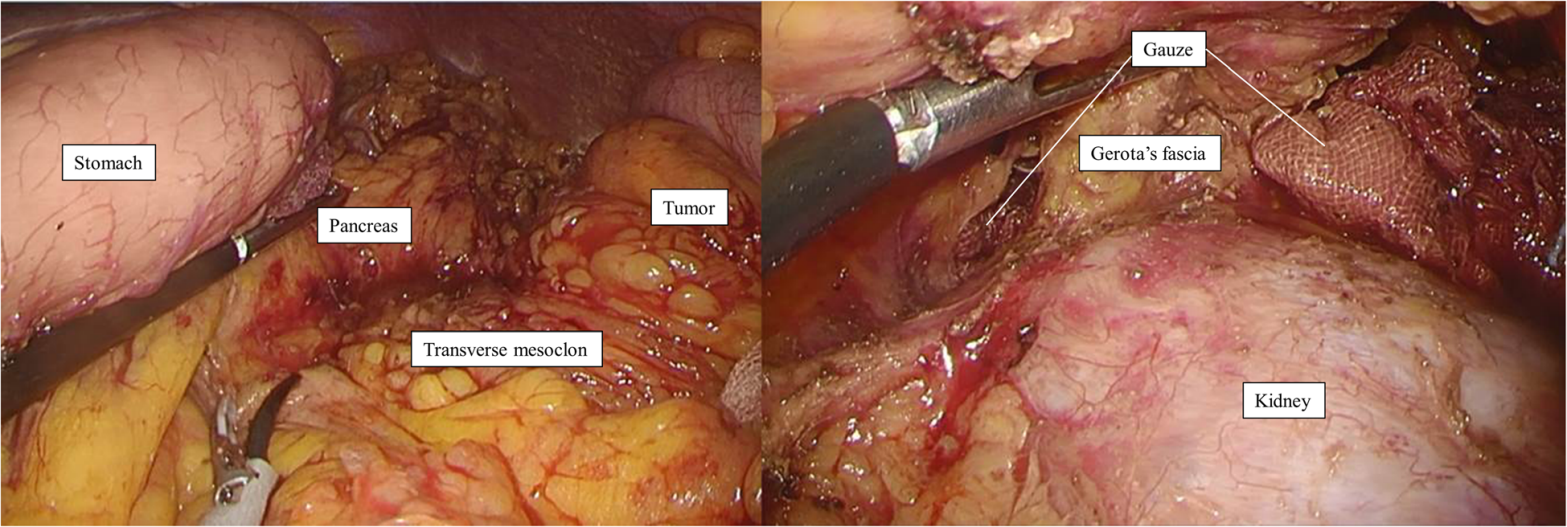
Laparoscopic cranial view of setting a “Goal” at the inferior border of pancreas and medial view of the resection of the Gerota’s fascia

The patient was placed in the lithotomy position under general and epidural anesthesia. The following ports were placed: a 12-mm port at the umbilicus for a scope and 5-mm ports at each quadrant. A fist-size tumor covered with the omentum was detected at the anal side of the splenic flexure. No peritoneal metastasis was observed:

1. Cranial approach to set a “Goal” for the medial approach
  First, the omental bursa was opened widely, and the adhesion between the transverse mesocolon and gastric posterior wall was dissected to visualize the pancreatic outline. The superior lobe and the fat of the transverse mesocolon were dissected in the proximity of the pancreas, and the posterior lobe of the transverse mesocolon was exposed cranially (Fig. 2). The gauze was placed on this layer, which was a “Goal” for the medial approach.
2. Mobilization of the left-sided colon and dissection of the regional nodes
  The sigmoid colon was mobilized in a caudal-to-cranial direction. After preserving the retroperitoneal tissue, including the hypogastric nerve plexus, ureter, and gonadal vessels, we exposed the root of the IMA clearly and dissected the swollen nodes along the IMA. The LCA was clipped and divided at the origin. Following this, the lateral attachment was dissected, and the descending colon was laterally mobilized to the splenic flexure.
3. Medial approach for safe resection of the Gerota’s fascia
  For obtaining a sufficient surgical margin, we dissected the Gerota’s fascia and exposed the surface of the left kidney just behind the tumor. At the most medial side of the mesocolon behind the IMV, we could easily detect the layer between the posterior lobe of the mesocolon and Gerota’s fascia; we cut the posterior lobe above the gauze that was placed as the “Goal” during the cranial approach. Finally, we cut the thick tissue, including the Gerota’s fascia and transverse mesocolon, just above the gauze (Fig. 2).

The mobilized left-sided colon was extracted through a trans-umbilical wound and dissected with sufficient proximal and distal margins. Functional end-to-end anastomosis was performed using linear staplers. The total operative time was 233 min, and the estimated total blood loss was 98 ml. She was discharged on the 14th day after surgery without postoperative complications.

**Fig. 3:**
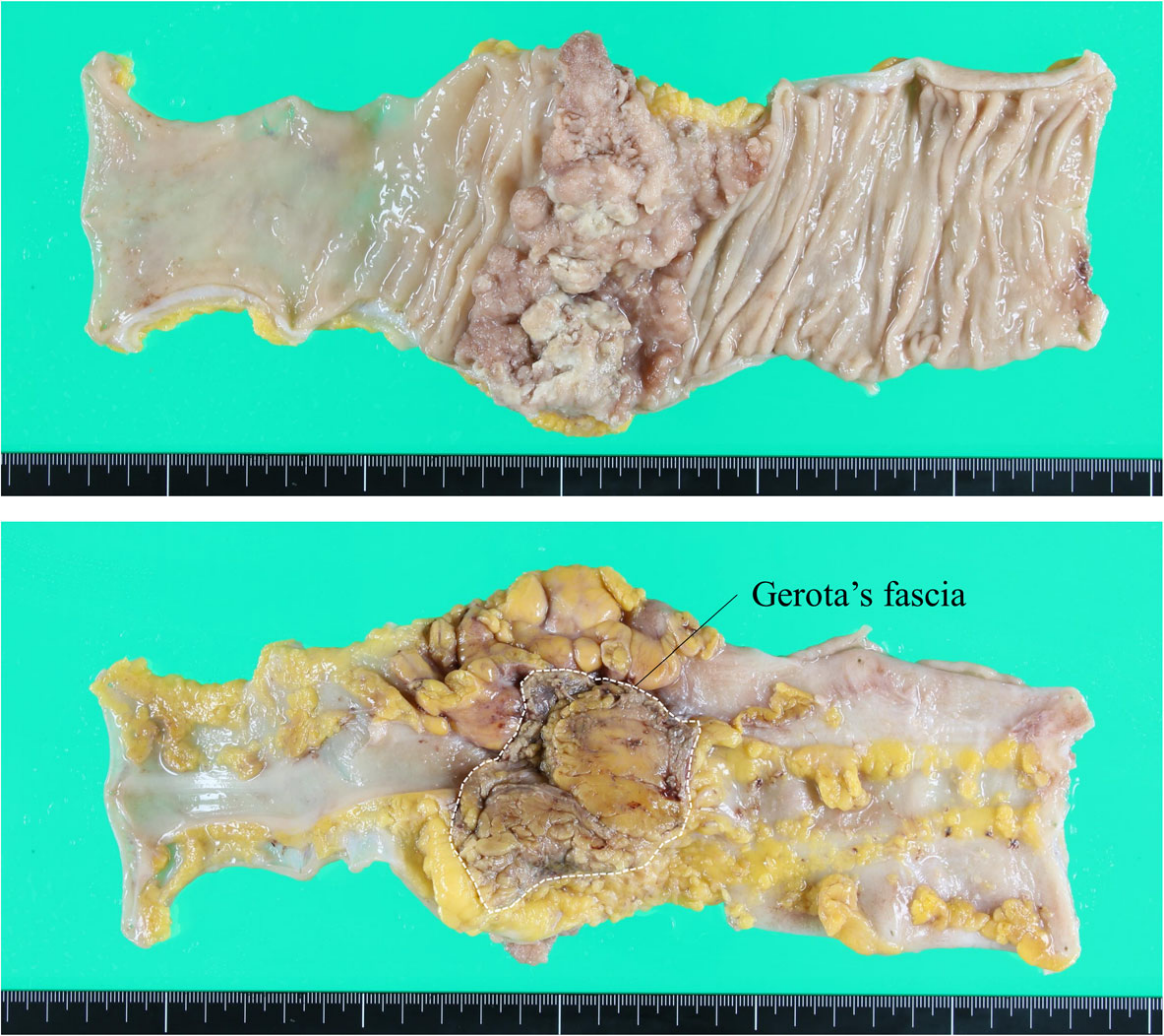
Resected specimens

Pathological findings revealed the invasion into the Gerota’s fascia, and the resection margin was negative for cancer (Fig. 3). The number of dissected lymph nodes was 18; however, no metastasis was observed. We assumed that the preoperative CT finding of lymphadenopathy was due to inflammation induced by the tumor. The pathological diagnosis was pT4bN0M0 pStage IIC.

## Discussion

This is the first case of cranial-first approach of laparoscopic left colectomy for T4 descending colon cancer invading the Gerota’s fascia. Previous studies have reported some advantages of laparoscopic surgery: good visualization, less blood loss, and less postoperative pain after colorectal cancer surgery [3–7]. However, the safety and feasibility of laparoscopic surgery for T4 colorectal cancer remain controversial [3, 8–10]. In terms of extended surgery for T4 colorectal cancer adhering to or invading neighboring organs, it would be more difficult to return from the deep layer to the shallow layer. Setting a “Goal” using a multidirectional approach of laparoscopic surgery can be the key to safe resection and can help minimize perioperative complications in patients with T4 colorectal cancer.

**Fig. 4:**
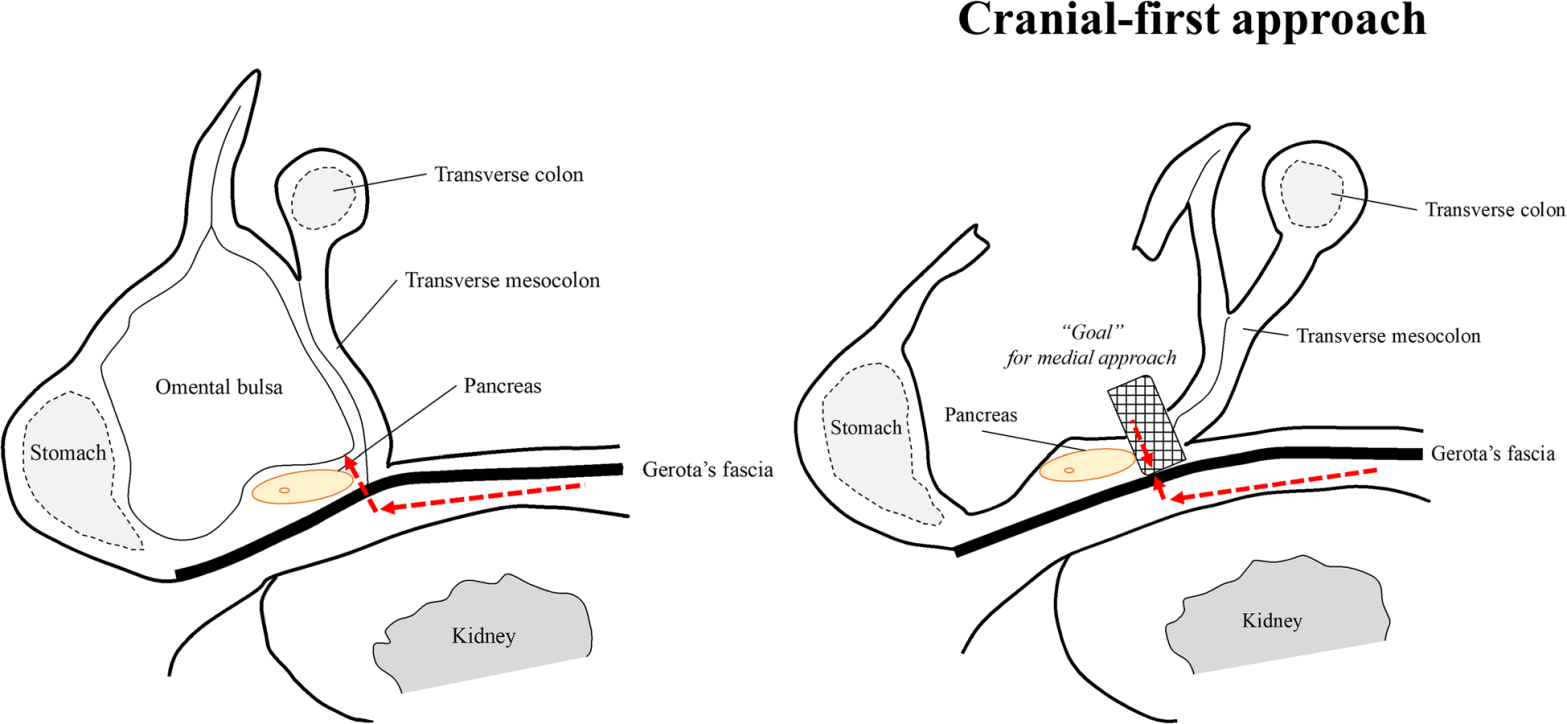
Scheme of cranial-first approach

For T4 descending colon cancer invading the Gerota’s fascia, dissection of the Gerota’s fascia behind the tumor is necessary for obtaining sufficient surgical margins. As shown in Fig. 4, the layer between the Gerota’s fascia and kidney is located below the pancreas. In the final part of the medial approach, the thick Gerota’s fascia and mesocolon backed by the pancreas often make dissection difficult due to the risk of pancreatic injury. The cranial-first approach, which we have reported previously, is a promising method for performing safe laparoscopic surgery with splenic flexure mobilization. Using this method of setting a “Goal” for the medial approach, we could easily achieve CME without pancreatic injury (Fig. 4). Owing to the complexity of distinguishing the layer to be dissected, this technique should be performed by laparoscopic surgeons with extensive experience in advanced colorectal cancer surgery.

## Conclusions

The cranial-first approach of laparoscopic left colectomy appears to be safe and feasible and could be a promising method for selected patients with T4 descending colon cancer invading the Gerota’s fascia.

## Data Availability

Data sharing is not applicable to this article as no datasets were generated or analyzed during the present study.

## Abbreviations

CME: Complete mesocolic excision
CT: Computed tomography
IMA: Inferior mesenteric artery
LCA: Left colic artery
